# Lung Metastasis From Uterine Leiomyosarcoma: A Case Report and Literature Review on Pulmonary Metastasis Presenting as Both Solid and Cystic Lesions

**DOI:** 10.7759/cureus.79484

**Published:** 2025-02-22

**Authors:** Amadora Li En Choo, Cynthia Ming Li Chia

**Affiliations:** 1 General Surgery, Singapore General Hospital, Singapore, SGP; 2 Cardiothoracic Surgery, Singapore General Hospital, Singapore, SGP

**Keywords:** lung metastasis, malignancy-associated pneumothorax, pneumothorax, pulmonary metastasis, uterine leiomyosarcoma

## Abstract

Uterine leiomyosarcoma is a rare, aggressive tumor that arises from the uterine smooth muscle cells. It has a poor prognosis, and distant metastasis is common. While pulmonary metastasis often presents as solid nodules, it may occasionally present as cystic lesions that can result in pneumothorax. Here, we describe a case of a 66-year-old woman with recurrent pulmonary metastasis secondary to uterine leiomyosarcoma who had different presentations of the same pathology. She was first diagnosed with stage 1B pT1bN0M0 uterine leiomyosarcoma and underwent a laparotomy and total hysterectomy and bilateral salpingo-oophorectomy (THBSO) with pelvic and para-aortic lymph node dissections. She declined adjuvant chemotherapy and was on close surveillance. Four years later, she was found to have a right upper lobe solid nodule that was confirmed to be metastatic leiomyosarcoma on biopsy, and she underwent a wedge resection of the lesion. A year later, she developed a new left apical cystic lesion associated with moderate left hydropneumothorax. She underwent a wedge resection of this lesion with talc pleurodesis, and histology confirmed metastatic uterine leiomyosarcoma. We reviewed the existing literature and postulated potential mechanisms and risk factors for the development of malignancy-associated pneumothorax.

## Introduction

Uterine leiomyosarcoma is a rare, aggressive tumor arising from the uterine smooth muscle cells. It has an annual incidence of 0.64 per 100,000 women and accounts for 2%-5% of all uterine malignancies [1]. A total hysterectomy with or without bilateral salpingo-oophorectomy is the treatment of choice for disease confined to the uterus. Pelvic and para-aortic lymphadenectomies are not routinely performed unless the patient has evidence of retroperitoneal lymphadenopathy [2]. Compared to other uterine malignancies, leiomyosarcoma has a high propensity for local recurrence and distant metastasis [3]. Poor prognostic factors include a tumor size of more than 5 cm and a high mitotic index, although they are highly aggressive even with a mitotic count of less than 2/mm^2^ [4]. While the lungs are the most common site for distant metastasis, isolated pulmonary metastasis can still be resected, with an overall survival of 45% and 35% at five and 10 years, respectively [5]. As such, it is important to identify pulmonary metastasis early when the disease is still resectable.

Pulmonary metastases typically present as well-circumscribed solid nodules, often in the periphery of the lungs due to hematogenous spread. Cystic pulmonary metastases are rare, although they have been reported secondary to soft tissue sarcomas [6]. These cystic pulmonary lesions may rupture resulting in spontaneous pneumothorax. Here, we present a case of a patient with recurrent pulmonary metastasis secondary to uterine leiomyosarcoma, presenting first as a solid nodule and later as a cystic lesion with hemopneumothorax.

## Case presentation

We report the case of a 66-year-old woman with recurrent pulmonary metastasis secondary to uterine leiomyosarcoma. She first presented with post-menopausal bleeding and was diagnosed with stage 1B pT1bN0M0 uterine leiomyosarcoma. This meant that the tumor was more than 5 cm but still confined to the uterus without pelvic lymphadenopathy or distant metastasis. She underwent a laparotomy and total hysterectomy and bilateral salpingo-oophorectomy (THBSO) with pelvic and para-aortic lymph node dissections. She declined adjuvant chemotherapy and was on close surveillance with yearly imaging.

**Figure 1:**
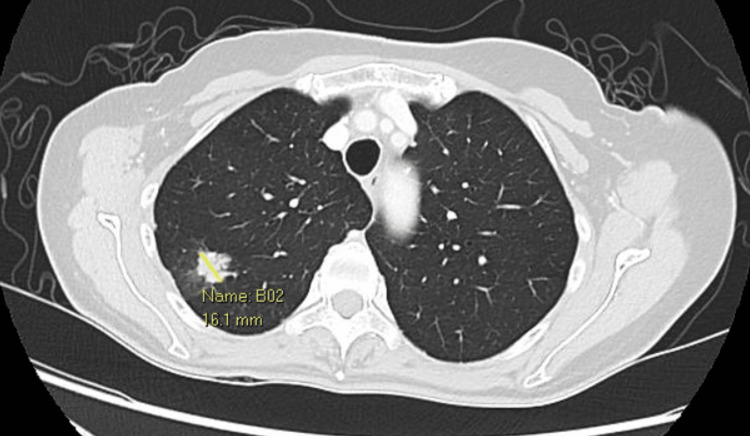
Axial cut of the chest CT showing a 1.6 cm solid nodule in the upper lobe of the right lung with perilesional ground glass changes, indeterminate for disease extension versus perilesional hemorrhagic change. This was consistent with biopsy-proven pulmonary metastasis secondary to uterine leiomyosarcoma. CT: computed tomography

Four years later, a computed tomography (CT) scan of the chest, abdomen, and pelvis revealed a new 1.1 cm solid nodule in the upper lobe of the right lung. An interval CT scan three months later showed a further increase in the size of this solid nodule from 1.1 cm to 1.6 cm, with perilesional ground glass changes (Figure 1), although the patient remained asymptomatic. A transthoracic needle biopsy of this lesion confirmed metastatic leiomyosarcoma. She underwent wedge resection of the right upper lobe lung lesion via a video-assisted thoracoscopic surgery (VATS) approach. Histopathological examination revealed a 1.3 cm focus of high-grade sarcoma, consistent with metastatic uterine leiomyosarcoma. Parenchymal resection margins were free of malignancy. She declined chemotherapy despite thorough counseling as she was concerned about the side effects and was thus followed up closely with yearly CT imaging.

**Figure 2:**
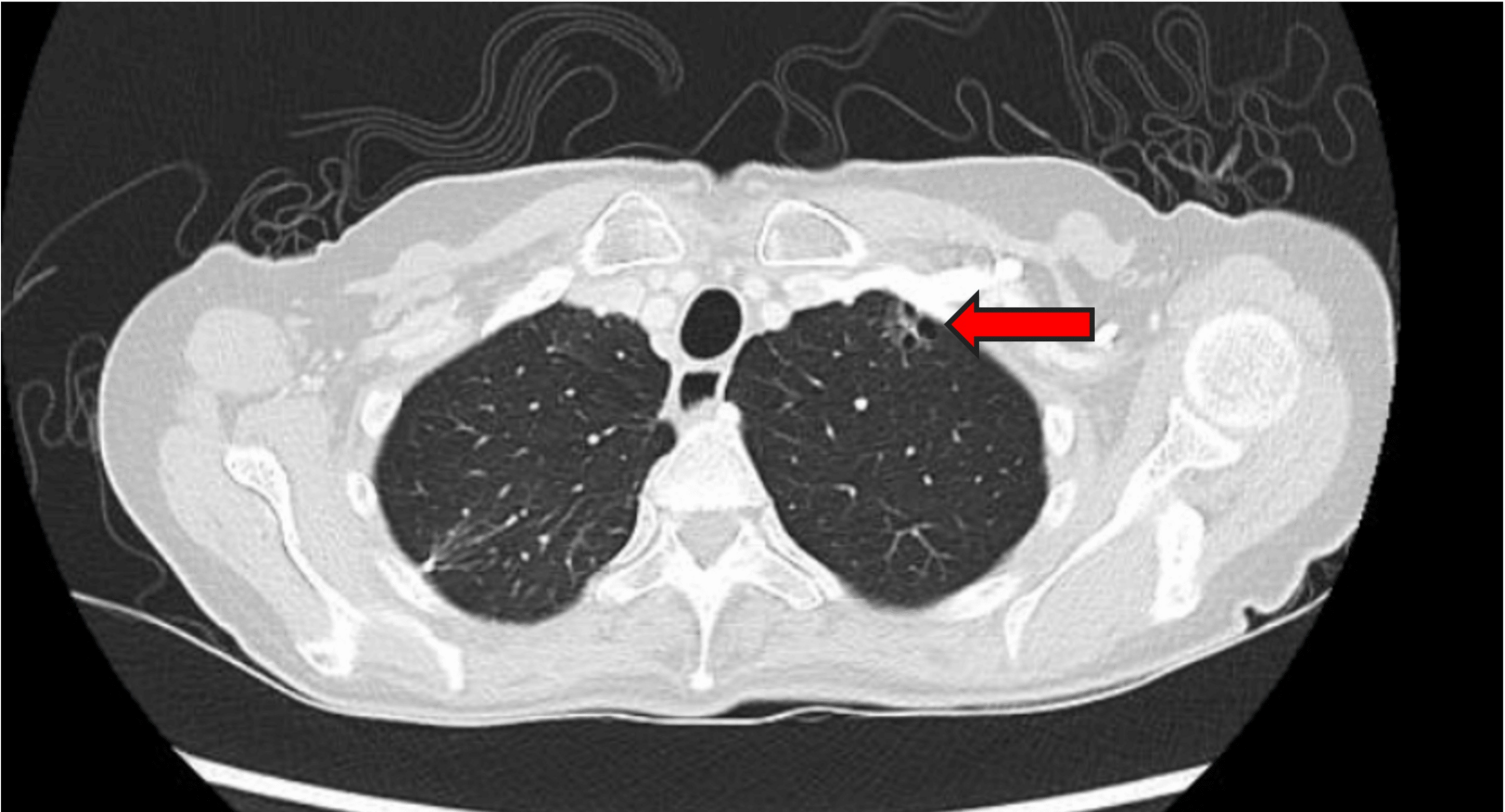
Axial cut of the chest CT showing a new 1 cm cystic focus at the left lung apex (red arrow). This occurred a year after the patient had a wedge resection of the right upper lobe pulmonary metastasis. CT: computed tomography

**Figure 3:**
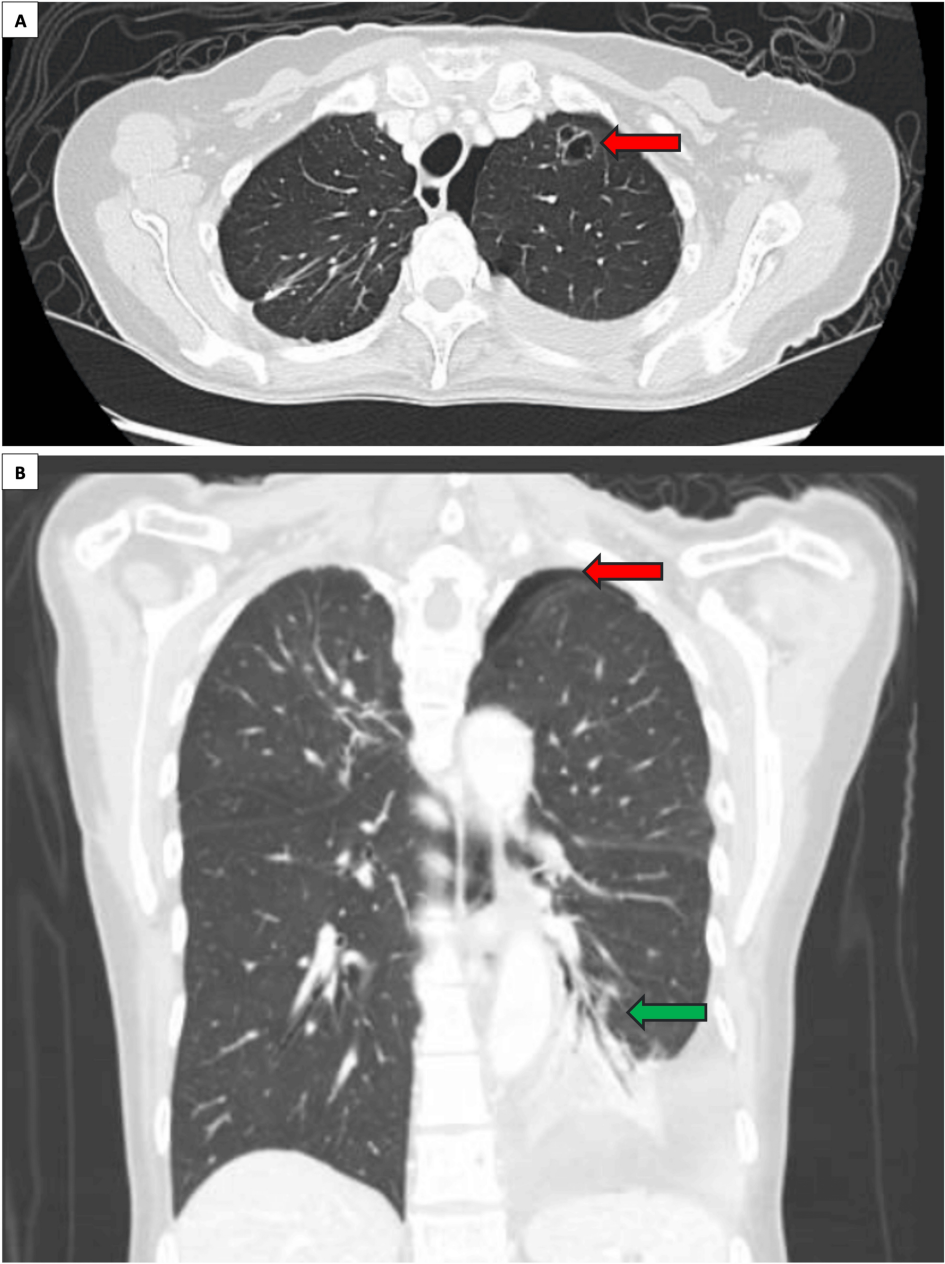
(A) Axial cut of the chest CT six months later, which reveals an interval increase in size of the left apical cystic lesion, from 1 cm to 1.9 cm (red arrow). (B) Coronal cut of the chest CT showing moderate left hydropneumothorax (red arrow) with partial collapse of the left lower lobe and adjacent compressive atelectasis (green arrow). CT: computed tomography

**Figure 4:**
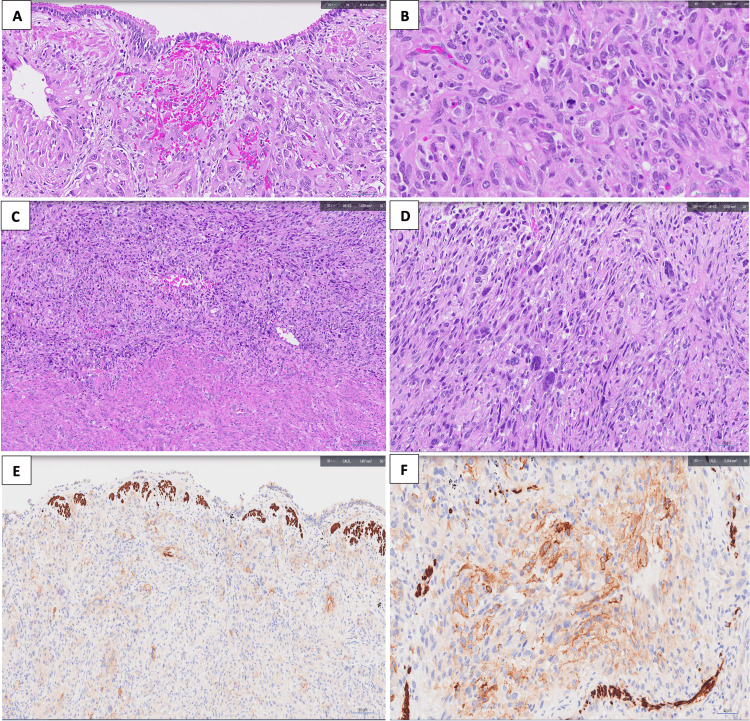
(A) Lung resection specimen (H&E stain, 200× magnification): pleomorphic epithelioid tumor with polygonal tumor cells with markedly enlarged nuclei and prominent nucleoli infiltrating the peribronchiolar parenchyma. (B) Lung resection specimen (H&E stain, 400× magnification): spindle and epithelioid features similar to those seen in the uterine tumor. (C) Hysterectomy specimen (H&E stain, 100× magnification): interface of the sarcoma with myometrium. (D) Hysterectomy specimen (H&E stain, 200× magnification): spindle and epithelioid features of the endometrial leiomyosarcoma. (E and F): Lung resection specimens at 200× magnification showing moderate to strong reaction to SMA and caldesmon immunohistochemical staining, respectively. H&E: hematoxylin and eosin

A year later, a routine CT scan of the chest revealed a new 1 cm left apical cystic focus (Figure 2). The patient was asymptomatic, and a decision was made to continue close surveillance. A CT scan six months later showed an interval increase in the size of the left apical bullae from 1 cm to 1.9 cm, associated with new moderate left hydropneumothorax (Figure 3). No other lesions were seen in the rest of the chest, abdomen, or pelvis, and the patient did not report any respiratory symptoms. A left chest tube was inserted, which confirmed the presence of hemopneumothorax. In view of the persistent air leak, a decision was made to proceed with surgery. She underwent wedge resection of the left upper lobe lesion via VATS approach with talc pleurodesis [7]. She recovered well postoperatively with no further air leak and was discharged on postoperative day 4. Histopathological examination of the resected lung specimen revealed an 8 mm high-grade pleomorphic sarcoma consistent with metastasis from uterine leiomyosarcoma. The overlying pleura was focally disrupted; however, broncho-vascular and parenchymal resection margins were negative for malignancy. Figure 4 demonstrates the similarities in histology between the lung resection and original leiomyosarcoma specimens, where both demonstrate spindle and epithelioid features. The lung tumor also stains moderate to strong for SMA and caldesmon on immunohistochemistry, consistent with uterine leiomyosarcoma.

Postoperatively, our patient still declined chemotherapy and was put on close surveillance. A CT scan six months later showed a 2 cm gastric body nodule, which was biopsied and found to contain atypical spindle cells. She underwent wedge resection of the gastric body tumor, and histology confirmed metastatic uterine leiomyosarcoma. She eventually agreed for systemic therapy and is currently on palliative docetaxel and gemcitabine.

## Discussion

Malignancy-related spontaneous pneumothorax is rare, accounting for 0.05%-1% of all spontaneous pneumothoraces [8]. Its prevalence is estimated to be 0.32% in patients with primary lung cancers [9] and 1.9% in patients with soft tissue sarcomas [10]. In 1937, De Barrin reported the first case of spontaneous hemopneumothorax in a patient with metastatic osteosarcoma [11]. Since then, there have been several case reports of patients with spontaneous pneumothorax secondary to sarcomatous pulmonary metastasis. A review of 189 patients by Ezzeddine and Jalal in 2017 showed that osteogenic sarcoma (31.7%), angiosarcoma (17.5%), and synovial cell sarcoma (11.1%) were the more commonly reported histologies [8]. These findings were similar to another review by Hoag et al. in 2010 [10].

Our literature search identified eight case reports of spontaneous pneumothorax secondary to pulmonary metastasis from uterine leiomyosarcoma (Table 1) [12-19]. Five patients presented with bilateral pneumothorax, while three had unilateral pneumothorax. Interestingly, four patients developed pneumothorax while on chemotherapy, with two of them developing it just after the first cycle of chemotherapy, when the lung metastasis itself had reduced in size. This is in keeping with one of the proposed mechanisms of malignancy-associated pneumothorax suggested by Thornton and Bigelow, where rapid necrosis of a subpleural nodule, often following chemotherapy, results in a bronchopleural fistula [20]. Direct invasion of the tumor into the pleura can also result in the development of pneumothorax. Another proposed mechanism for the development of malignancy-associated pneumothorax is the rupture of a subpleural bleb. Pulmonary metastases may result in partial bronchiolar obstruction and a ball valve effect where air flows into the distal airspaces without outflow, causing increased pressure and the formation of thin-walled cysts and blebs that can eventually rupture, resulting in pneumothorax. Metastatic pulmonary nodules may also undergo a cystic transformation from tumor necrosis, either caused by aggressive tumor growth or chemotherapy, and the rupture of these cystic lesions can result in pneumothorax. Lastly, the case illustrated by Obeidat et al. (2023) describes another mechanism for the development of pneumothorax, where a large obstructing endobronchial mass results in lung collapse, creating a negative pressure space in the pleura and lung entrapment [12].

**Table 1:** Case reports of pulmonary metastasis from uterine leiomyosarcoma presenting with pneumothorax. THBSO: total hysterectomy and bilateral salpingo-oophorectomy

Reference	Age	Prior treatment	Presentation	Type of pneumothorax	Nature of pulmonary metastasis	Treatment for pneumothorax	Patient outcomes
Obeidat et al. (2023) [12]	48	-	Shortness of breath	Bilateral	Endobronchial mass causing left upper lobe collapse with bilateral pulmonary nodules	Bilateral chest tube insertion	Rapid progression of metastatic disease, persistent bilateral air leak, and respiratory failure requiring intubation; eventual decision for comfort care
Mocerino et al. (2018) [13]	40	THBSO, lymph node dissection, omentectomy, followed by chemotherapy	Fever, cough (ongoing chemotherapy)	Bilateral	Bilateral pulmonary nodules	Bilateral chest tube insertion, followed by talc pleurodesis on the right (patient refused left-sided pleurodesis as she wanted to return home)	Discharged after 27 days and restarted chemotherapy one week later
Murakami et al. (2016) [14]	52	THBSO, followed by chemotherapy	After one cycle of chemotherapy, lung metastasis reduced in size but developed bilateral pneumothorax	Bilateral	Bilateral pulmonary nodules	Talc pleurodesis	Lungs re-expanded and stabilized in seven days; resumed chemotherapy with no recurrence of pneumothorax
Alappan et al. (2013) [15]	55	THBSO, followed by chemotherapy	Right-sided chest pain, dyspnea (ongoing chemotherapy)	Unilateral	Bilateral pulmonary nodules with necrosis of some metastatic lesions	Not mentioned	Not mentioned
Fujiwara et al. (2011) [16]	57	THBSO, followed by chemotherapy and radiotherapy	Dyspnea (one year after chemotherapy)	Unilateral	Thin-walled cavitary nodule in the middle lobe and bilateral lower lobes	Chest tube insertion followed by thoracoscopic partial resection of the middle lobe and right lower lobe	Not mentioned
Mizushina et al. (2008) [17]	44	THBSO	CT findings	Unilateral	Multiple cystic lesions	Surgery	Not mentioned
Fenlon et al. (1996) [18]	57	THBSO, followed by chemotherapy	Dyspnea, pleuritic chest pain after one cycle of chemotherapy	Bilateral, recurrent	Bilateral pulmonary nodules	Chest tube insertion, followed by chemical pleurodesis, eventually had pleurectomy due to recurrent pneumothorax	Discharged home; four weeks later, both lungs remained fully expanded; continued on chemotherapy without complications
Mehzad (1977) [19]	20	-	Cyanosis, severe dyspnea	Bilateral	Bilateral subpleural cystic lesions	-	The patient was moribund on arrival at the hospital and demised from respiratory failure

Treatment of malignancy-associated pneumothorax may be difficult especially if the underlying mechanism is a bronchopleural fistula, which could lead to persistent air leak despite chest tube insertion and recurrent pneumothorax. In such situations, chemical pleurodesis via intrapleural instillation of talc or surgical resection should be considered.

## Conclusions

While pulmonary metastasis more often presents as a solid nodule, it may occasionally present with cystic lesions that can result in pneumothorax. In this case report, we described a patient with pulmonary metastasis secondary to uterine leiomyosarcoma who had different presentations of the same pathology, first presenting as a solid nodule and later as a cystic apical lesion with hemopneumothorax. We reviewed the existing literature for similar case reports and possible mechanisms for the development of malignancy-associated pneumothorax. We postulate that potential risk factors include subpleural cystic lesions and tumors that undergo rapid necrosis following good response to chemotherapy, although future studies should be undertaken to validate this hypothesis. On a more practical note, this case highlights that cystic lung lesions can also represent metastatic malignancy, especially in patients with a history of soft tissue sarcoma, and it is important to bear this in mind during surveillance imaging or when a patient presents with spontaneous pneumothorax.
